# Seasonality of respiratory syncytial virus infection in children hospitalized with acute lower respiratory tract infections in Hunan, China, 2013–2022

**DOI:** 10.1186/s12985-024-02336-8

**Published:** 2024-03-07

**Authors:** Le-Yun Xie, Tao Wang, Tian Yu, Xian Hu, Le Yang, Li-Li Zhong, Bing Zhang, Sai-Zhen Zeng

**Affiliations:** Hunan Provincial People’s Hospital (The First Affiliated Hospital of Hunan Normal University), 410005 Changsha, China

**Keywords:** Respiratory syncytial virus, Seasonality, Children, COVID-19

## Abstract

**Background:**

In China, respiratory syncytial virus (RSV) infections traditionally occur during the spring and winter seasons. However, a shift in the seasonal trend was noted in 2020–2022, during the coronavirus disease 2019 (COVID-19) pandemic.

**Methods:**

This study investigated the seasonal characteristics of RSV infection in children hospitalized with acute lower respiratory tract infections (ALRTIs). The RSV epidemic season was defined as RSV positivity in > 10% of the hospitalized ALRTI cases each week. Nine RSV seasons were identified between 2013 and 2022, and nonlinear ordinary least squares regression models were used to assess the differences in year-to-year epidemic seasonality trends.

**Results:**

We enrolled 49,658 hospitalized children diagnosed with ALRTIs over a 9-year period, and the RSV antigen-positive rate was 15.2% (*n* = 7,566/49,658). Between 2013 and 2022, the average onset and end of the RSV season occurred in week 44 (late October) and week 17 of the following year, respectively, with a typical duration of 27 weeks. However, at the onset of the COVID-19 pandemic, the usual spring RSV peak did not occur. Instead, the 2020 epidemic started in week 32, and RSV seasonality persisted into 2021, lasting for an unprecedented 87 weeks before concluding in March 2022.

**Conclusions:**

RSV seasonality was disrupted during the COVID-19 pandemic, and the season exhibited an unusually prolonged duration. These findings may provide valuable insights for clinical practice and public health considerations.

**Supplementary Information:**

The online version contains supplementary material available at 10.1186/s12985-024-02336-8.

## Background

Globally, respiratory syncytial virus (RSV) is a prevalent cause of acute lower respiratory tract infections (ALRTIs) in childhood, and contributes significantly to hospital admissions among young children. This places a substantial burden on healthcare services. Nearly half of the worldwide disease burden associated with RSV occurs in just five countries: Pakistan, India, Nigeria, Indonesia, and China. In China, the estimated annual hospitalizations for infants and young children due to RSV infection range from 215,000 to 500,000 [1].

RSV activity exhibits a seasonal pattern in most regions, and its seasonal epidemics are a leading cause of hospitalization and mortality globally, particularly due to bronchiolitis and pneumonia [2, 3]. The RSV season is typically characterized by an RSV rate exceeding the defined threshold for a specific duration. RSV infection incidence peaks during winter and spring in temperate regions and during rainy seasons in tropical regions. The RSV season commences between March and June in countries in the Southern hemisphere and between September and December in the Northern hemisphere [4]. A study reported the long-term time-series data of medically attended first-time RSV infection among young children. From 2010 to 2019, the monthly incidence rate of medically attended RSV infection in children aged 0–5 years of the United States followed a consistent seasonal pattern: rising from September to November, peaking from December to January, then dropping from February to April, with sustained low rate during May to August [5]. Another study found that states with colder, drier weather and a large seasonal swing in potential evapotranspiration tended to experience an alternating pattern of ‘‘early-big’’ RSV epidemics one year followed by a ‘‘late-small’’ epidemic the next year [6]. In China, the incidence of RSV infection typically peaks between November and February of the following year [7].

Several RSV vaccine candidates and monoclonal antibodies are currently in the advanced clinical development stage [8]. Therefore, prevention of RSV transmission remains a promising strategy to control seasonal epidemics. Prior to the COVID-19 pandemic, RSV epidemiology adhered to a seasonal pattern worldwide [3]. Interestingly, the COVID-19 pandemic has significantly influenced RSV epidemiology, with many countries experiencing an absence of RSV infections during the first pandemic winter [9]. A delayed summer epidemic was observed in various locations worldwide [10–12]. The success in preventing RSV infections was attributed to the strict implementation of non-pharmacological public health interventions targeting COVID-19. Concerns have been raised regarding potential severe RSV epidemics in the future due to “immunity debt,” a term that describes reduced protective immunity resulting from prolonged periods of low exposure to a pathogen, rendering a greater proportion of the population susceptible to the disease [13, 14].

China bears a significant burden of RSV infection [15], but few studies have assessed the seasonality or trends of RSV infections. Furthermore, during the COVID-19 outbreak, limited availability of epidemiological surveillance data for other respiratory viruses could have impeded the implementation of therapy and prophylactic interventions for RSV. The present study retrospectively examined available surveillance data for RSV in children hospitalized with ALRTI in Hunan, China, between 2013 and 2022. It evaluated seasonal changes in RSV infections both before and after the COVID-19 pandemic.

## Methods

### Population and methods

This retrospective study spanned a 9-year period from July 1, 2013, to June 30, 2022. All children hospitalized with ALRTI were included in the RSV epidemiological surveillance program conducted at the Children’s Medical Center of Hunan Provincial People’s Hospital (The First Affiliated Hospital of Hunan Normal University). Samples were collected after obtaining informed consent from the parents or guardians of each child, and the research protocol received approval from the hospital’s ethics review committee.

The collected data included the date of hospital admission, demographic information, disease severity, length of hospital stay, and the cost of stay in pediatric wards. Recorded complications included congenital heart disease, malnutrition, premature birth, chronic lung disease, anemia, and asthma. We collected China’s GDP per habitant in the study period to compare the cost with an average outcome (2013, US $7020; 2014, US $7636; 2015, US $8016; 2016, US $8094; 2017, US $8817; 2018, US $9905; 2019, US $10,143; 2020, US $10,408; 2021, US $12,617; and 2022, US $12,720, respectively).

#### Experimental process

Nasopharyngeal swabs were obtained within 24 h of hospitalization for virological diagnosis. Nasopharyngeal aspirate specimens of the enrolled children were collected by trained nurses after admission and were transported immediately to the clinical laboratory center. Seven common pathogens included respiratory syncytial virus (RSV), adenovirus (ADV), influenza virus A (Flu A), influenza virus B (Flu B), and parainfluenza virus types 1–3 (PIV1–3). For the DFA, the cell pellets from the NPS samples were suspended in several drops of sterile phosphate-buffered saline, and the resulting cell suspension was spotted onto an acetone-cleaned slide. An anti-RSV monoclonal antibody labeled with fluorescein isothiocyanate from the D3 UltraTM DFA Respiratory Virus Screening & ID Kit (Diagnostic Hybrids Inc., Athens, OH, USA) was used for RSV identification using the DFA and was conducted by professional staff following standard operating procedures.

#### Definitions

The “monitoring year” was defined as July 1 (week 27) to June 30 of the subsequent year. All enrolled hospitalized patients were 14 years of age or younger, admitted for ALRTI, and diagnosed based on clinical and radiologic findings. Eligible children exhibited an illness characterized by an acute or worsened cough as the primary or dominant symptom, or lower respiratory tract infection symptoms lasting less than 28 days. Exclusion criteria were children with immunosuppression related to solid organ or hematopoietic stem cell transplantation, chemotherapy, tumors, hematological diseases, a history of HIV, steroid treatment for more than 30 days, or immunosuppressant treatment.

RSV positivity rate was defined as the number of positive RSV specimens divided by the total number of specimens, multiplied by 100. Seasons were categorized as winter (weeks 49 to 9 of the following year), spring (weeks 10–22), summer (weeks 23–35), and autumn (weeks 36–48). Five age groups were defined: 28 days to 5 months, 6–11 months, 12–23 months, 24–59 months, and ≥ 60 months.

The RSV season was defined as consecutive weeks during which the percentage of RSV testing positive per week exceeded a 10% threshold [7, 16–18]. The onset week was the first of two consecutive weeks when the weekly percentage of specimens testing positive for RSV was ≥ 10%, with at least 20 specimens tested per week. The offset week was the last of two consecutive weeks when the weekly percentage of specimens testing positive for RSV was ≥ 10%, without any gap weeks.

### Statistical analysis

Data were compiled using Excel 2016 software (Microsoft Corp., Redmond, WA, USA) and analyzed using R software (version 3.5.2). Count data are presented as percentages, and group comparisons were conducted using the χ^2^ test. The Kruskal-Wallis test was used to analyze non-normally distributed data. *P*-values < 0.05 were considered statistically significant.

Based on the US Centers for Disease Control and Prevention guidelines, the RSV season was defined as the period during which the weekly rate of RSV-positive tests exceeded 10% for several consecutive weeks [7, 16–18]. A logistic regression model (incorporating sine and cosine functions of the illness onset week) was fitted to individual patient data, preserving a seasonal curve as previously described [19]. Python software and nonlinear least square curve fitting were utilized for fitting a cosine curve.

## Results

### Patient characteristics

**Fig. 1:**
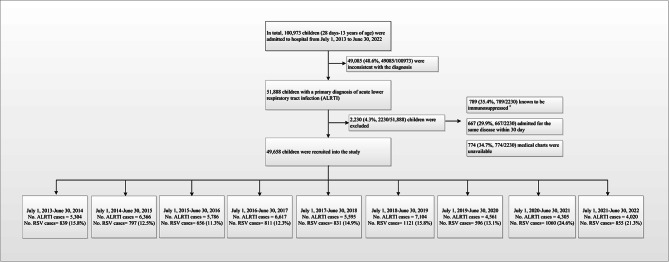
Flow chart for the enrollment of pediatric ALRTI inpatients for detection of RSV in July 1, 2013 - June 30, 2022 study. #: immunosuppressed was defined as having received a solid organ or hematopoietic stem cell transplant, undergoing chemotherapy, having a history of human immunodeficiency virus, or using steroids for > 30 days. Abbreviation: ALRTI, acute lower respiratory tract infection; RSV, respiratory syncytial virus

Between July 1, 2013, and June 30, 2022, 49,658 hospitalized children diagnosed with ALRTI were included in the study (Fig. 1). Among them, 30,413 were males (61.2%) and 19,245 were females (38.8%), with a male-to-female ratio of approximately 1.6:1. The age range of the children was 1–168 months, with a median age of 16 months (interquartile range [IQR]: 6–38 months). Children aged < 5 years constituted 90.2% of the study sample. The median length of hospital stay was 6 days (IQR: 5–8 days), and the median cost of hospitalization was 961 dollars (IQR: 770–1,318 dollars). A significant decline in the number of hospitalized ALRTI patients occurred after the severe acute respiratory syndrome coronavirus 2 (SARS-CoV-2) outbreak in China in February 2020, coupled with the implementation of non-pharmaceutical interventions (NPIs). In February, March, and April 2020, there were 52, 56, and 39 ALRTI-related hospitalizations, compared to 659, 744, and 660 cases in the corresponding months in 2019, respectively. This marked a decrease of 92.1%, 92.4%, and 94.1%, respectively. During 2019–2020, 2020–2021, and 2021–2022, the number of ALRTI hospitalizations decreased significant (*P* < 0.01) compared to 2018–2019, with reductions of 36%, 39%, and 43%, respectively (Table 1).

**Table 1:** Demographic characteristics of hospitalized children with pneumonia, Hunan, China, July 1, 2013-June 30, 2022

Characteristics	2013–2014 No. (%)	2014–2015 No. (%)	2015–2016 No. (%)	2016–2017 No. (%)	2017–2018 No. (%)	2018–2019 No. (%)	2019–2020 No. (%)	2020–2021 No. (%)	2021–2022 No. (%)	Total	χ2 /H	*P* value	
No. patients	5304	6366	5786	6617	5595	7104	4561	4305	4020	49,658			
Gender, male	3384 (63.8)	3989 (62.7)	3551 (61.4)	4080 (61.7)	3463 (61.9)	4282 (60.3)	2690 (59.0)	2596 (60.3)	2378 (59.2)	30,413 (61.2)	43.189	<0.001	
Age, months	13 (6, 30)	13 (6, 33)	14 (6, 36)	13 (5, 35)	13 (6, 35)	16 (7, 38)	24 (10, 44) ^a^	20 (9, 40) ^b^	28 (10, 48) ^c^	16 (6, 38)	1311.484	<0.001	
Age group, months											1616.005	<0.001	
1–5	1199 (22.6)	1590 (25.0)	1317 (22.8)	1820 (27.5)	1302 (23.3)	1480 (20.8)	690 (15.1)	651 (15.1)	534 (13.3)	10,583 (21.3)			
6–11	1223 (23.1)	1313 (20.6)	1233 (21.3)	1258 (19.0)	1167 (20.9)	1335 (18.8)	664 (14.6)	693 (16.1)	610 (15.2)	9496 (19.1)			
12–23	1197 (22.6)	1319 (20.7)	1138 (19.7)	1243 (18.8)	1222 (21.8)	1522 (21.4)	861 (18.9)	1010 (23.5)	669 (16.6)	10,181 (20.5)			
24–59	1303 (24.6)	1682 (26.4)	1613 (27.9)	1720 (26.0)	1361 (24.3)	1961 (27.6)	1719 (37.7)	1639 (38.1)	1525 (37.9)	14,523 (29.2)			
≥ 60	382 (7.2)	462 (7.3)	485 (8.4)	576 (8.7)	543 (9.7)	806 (11.3)	627 (13.7)	312 (7.2)	682 (17.0)	4875 (9.8)			
LOS	7 (6, 9)	7 (5, 8)	6 (5, 8)	6 (5, 8)	6 (5, 8)	6 (5, 7)	6 (5, 7)	5 (5, 7)	6 (5, 7)	6 (5, 8)	1746.879	<0.001	
Hospital costs, $	894 (722, 1185)	986 (792, 1337)	967 (783, 1298)	925 (744, 1250)	999 (797, 1383)	977 (769, 1408)	914 (741, 1236)	922 (750, 1234)	1071 (866, 1559)	961 (770, 1318)	843.718	<0.001	
Admission into PICU, days	184 (3.5)	355 (5.6)	369 (6.4)	437 (6.6)	475 (8.5)	600 (8.4)	153 (3.4)	156 (3.6)	142 (3.5)	2871 (5.8)	308.732	<0.001	
Severe pneumonia	382 (7.2)	498 (7.8)	381 (6.6)	457 (6.9)	492 (8.8)	712 (10.0)	209 (4.6)	190 (4.4)	181 (4.5)	3502 (7.1)	201.124	<0.001	

Note: The data of age, months, LOS, and hospital costs were presented with a median (P25, P75). No. (%), which stands for patient number (percentage). LOS means length of stay. PICU means pediatric intensive care unit. $ means US dollar. ^a^*P* < 0.05, for comparison of median age in 2019–2020 with other groups; ^b^*P* < 0.05, for comparison of median age in 2020–2021 with other groups; ^c^*P* < 0.05, for comparison of median age in 2021–2022 with other groups (exclude 2020–2021)

### Clinical and epidemiological characteristics of RSV-positive cases

**Fig. 2:**
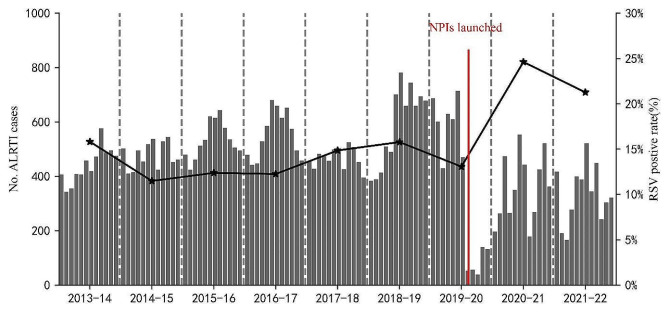
Cases of ALRTI in hospitalized children 28 days–13 years of age, by month, Hunan, China, July 1, 2013 - June 30, 2022. The vertical grey dashed line marked the separation of two study years. The line graphs marked RSV positive rate by year. The vertical red solid line, as the dividing line, marked the highest emergency response to COVID-19, was launched, in Hunan. Abbreviation: ALRTI, acute lower respiratory tract infection; RSV, respiratory syncytial virus; NPIs, non-pharmaceutical interventions

**Fig. 3:**
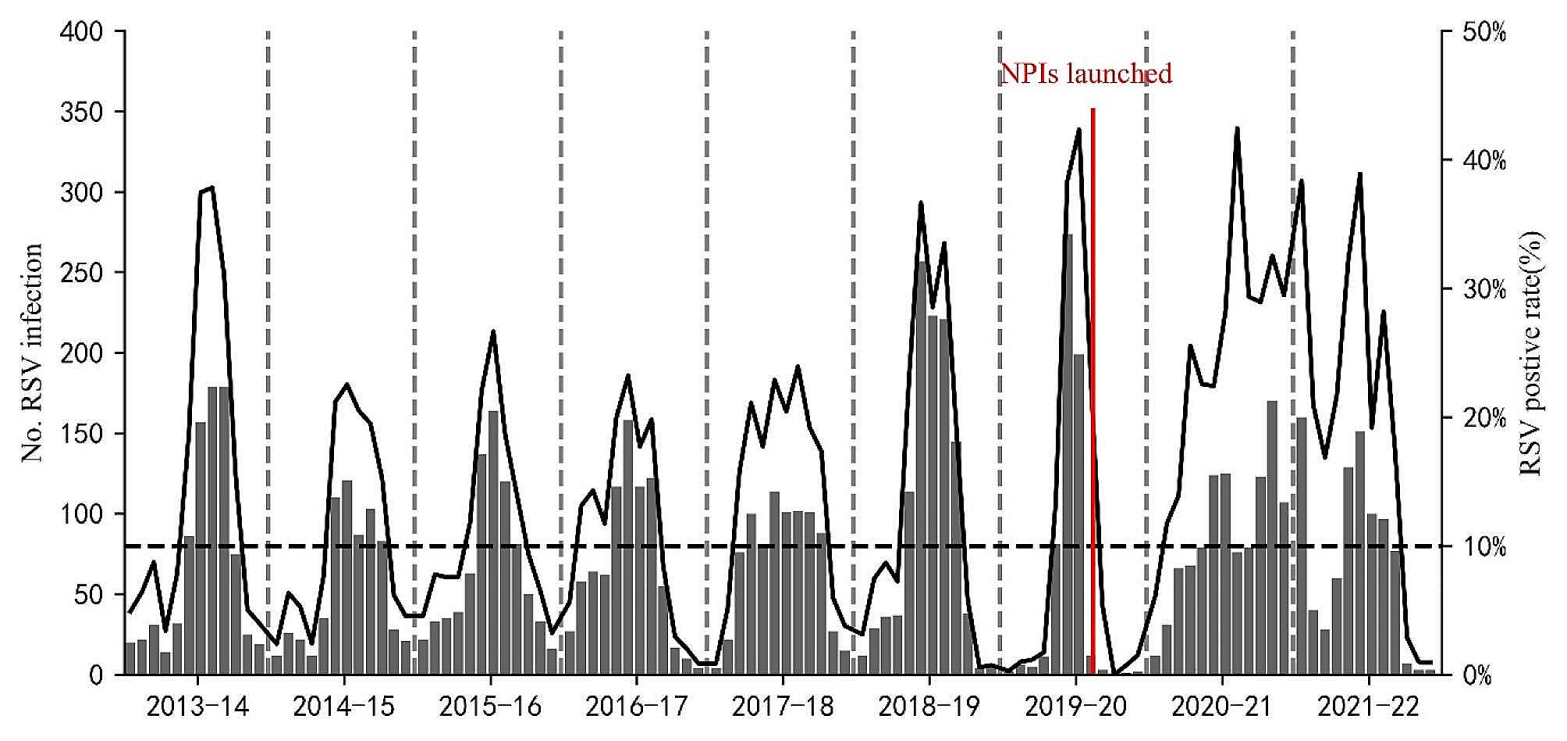
Monthly distribution of RSV-positive specimens in children with ALRTI in Hunan from July 1, 2013 to June 30, 2022. The numbers of RSV-positive specimens are shown in the grey column diagram, and the positive rates of RSV are shown in the black line graph. Abbreviation: ALRTI, acute lower respiratory tract infection; RSV, respiratory syncytial virus

The RSV antigen-positive rate was 15.2% (*n* = 7,566/49,658), with 4,805 cases among boys and 2,761 among girls. The median age of RSV-positive patients was 9 months (IQR: 4–20 months), with 33.8% (*n* = 2,556/7,566) of the patients aged < 6 months, and the RSV-positive rate gradually decreased with increasing age. Among RSV-positive patients, 16.3% (*n* = 1,236/7,566) presented with a concurrent disease or condition upon admission. The most common conditions included prematurity (*n* = 523/7,566; 6.9%), congenital heart disease (*n* = 356/7,566; 4.7%), asthma (*n* = 220/7,566; 2.9%), anemia (*n* = 97/7,566; 1.3%), chronic lung disease (*n* = 28/7,566; < 1%), and malnutrition (*n* = 12/7,566; < 1%) (Table 2). The RSV-positive rate also declined significantly after the SARS-CoV-2 outbreak in China in February 2020. In February, March, and April 2020, there were 12, 3, and 0 RSV-positive cases, compared to 221, 145, and 38 cases, in 2019, respectively, marking decreases of 94.6%, 97.9%, and 100%, respectively (Table 2; Figs. 2 and 3). The epidemiology of the other virus infection in the enrolled patients was present in supplementary Table 1. Other virus co-infections with RSV were present in supplementary Table 2.

**Table 2:** Demographic characteristics of RSV positivity children hospitalized with acute lower respiratory tract infection in Hunan, China, July 1, 2013–June 30, 2022

Characteristics	2013–2014 No. (%)	2014–2015 No. (%)	2015–2016 No. (%)	2016–2017 No. (%)	2017–2018 No. (%)	2018–2019 No. (%)	2019–2020 No. (%)	2020–2021 No. (%)	2021–2022 No. (%)	Total	χ^2^/H		*P*
No. patients	839	797	656	811	831	1121	596	1060	855	7566			
Gender, male	551 (65.7)	516 (64.7)	402 (61.3)	519 (64.0)	547 (65.8)	710 (63.3)	368 (61.7)	637 (60.1)	555 (64.9)	4805 (63.5)	12.504		0.130
Age, months	8.0 (4.0, 16.0)	7.0 (3.0, 14.0)	7 (3, 15)	7 (3, 15)	8 (4, 14)	8 (3, 16)	12 (5, 28)^a^	14 (7, 29)^b^	15 (6, 31)^c^	9(4, 20)	526.252		<0.001
Age group, months											542.017		<0.001
1–5	295 (35.2)	336 (42.2)	264 (40.2)	372 (45.9)	301 (36.2)	424 (37.8)	168 (28.2)	215 (20.3)	181 (21.2)	2556 (33.8)			
6–11	239 (28.5)	223 (28.0)	179 (27.3)	175 (21.6)	246 (29.6)	282 (25.2)	128 (21.5)	221 (20.8)	180 (21.1)	1873 (24.8)			
12–23	190 (22.6)	138 (17.3)	121 (18.4)	137 (16.9)	167 (20.1)	241 (21.5)	112 (18.8)	276 (26.0)	192 (22.5)	1574 (20.8)			
24–59	114 (13.6)	95 (11.9)	87 (13.3)	120 (14.8)	113 (13.6)	165 (14.7)	180 (30.2)	334 (31.5)	283 (33.1)	1491 (19.7)			
≥ 60	1 (0.1)	5 (0.6)	5 (0.8)	7 (0.9)	4 (0.5)	9 (0.8)	8 (1.3)	14 (1.3)	19 (2.2)	72 (1.0)			
Underlying medical condition													
CHD#	49 (5.8)	54 (6.8)	36 (5.5)	47 (5.8)	44 (5.3)	41 (3.7)	12 (2.0)	37 (3.5)	36 (4.2)	356 (4.7)	30.048		<0.001
Chronic lung diseases*	4 (0.5)	2 (0.3)	4 (0.6)	4 (0.5)	3 (0.4)	2 (0.2)	2 (0.3)	4 (0.4)	3 (0.4)	28 (0.4)	3.069		0.930
Anemia (Hb<90 g/l)†	16 (1.9)	12 (1.5)	12 (1.8)	12 (1.5)	7 (0.8)	16 (1.4)	2 (0.3)	12 (1.1)	8 (0.9)	97 (1.3)	11.380		0.181
Malnutrition	2 (0.2)	2 (0.3)	1 (0.2)	3 (0.4)	2 (0.2)	1 (0.1)	0 (0.0)	1 (0.1)	0 (0.0)	12 (0.2)	6.331		0.610
Asthma	26 (3.1)	15 (1.9)	16 (2.4)	16 (2.0)	20 (2.4)	36 (3.2)	23 (3.9)	36 (3.4)	32 (3.7)	220 (2.9)	12.122		0.146
History of prematurity&	22 (2.6)	56 (7.0)	48 (7.3)	37 (4.6)	52 (6.3)	116 (10.3)	12 (2.0)	102 (9.6)	78 (9.1)	523 (6.9)	93.081		<0.001
LOS	7 (6, 9)	7 (6, 9)	7 (5, 8)	6 (5, 8)	6 (5, 8)	6 (5, 8)	6 (5, 7)	6 (5, 7)	6 (5, 7)	6 (5, 8)	419.400		<0.001
hospital costs, $	920 (767, 1173)	1072 (853, 1480)	1032 (835, 1438)	967 (794, 1311)	1094 (853, 1446)	975 (771, 1382)	856 (726, 1083)	1000 (791, 1395)	1052 (855, 1501)	992 (799, 1362)	239.508		<0.001
Admission into PICU	34 (4.1)	82 (10.3)	59 (9.0)	70 (8.6)	85 (10.2)	115 (10.3)	8 (1.3)	49 (4.6)	72 (8.4)	574 (7.6)	93.330		<0.001
Severe pneumonia	91 (10.8)	118 (14.8)	92 (14.0)	79 (9.7)	107 (12.9)	151 (13.5)	26 (4.4)	74 (7.0)	89 (10.4)	827 (10.9)	72.214		<0.001
Co-infected	51(6.1)	68(8.5)	67(10.2)	53(6.5)	79(9.5)	140(11.3)	26(4.4)	18(1.7)	36(4.2)	525(6.9)	118.205		<0.001

Notes: The data of age, months, LOS, and hospital costs were presented with a median (P25, P75). No. (%), which stands for patient number (percentage). LOS means length of stay. PICU means pediatric intensive care unit. $ means US dollar. &Prematurity was defined as birth at gestational age < 37 weeks. #CHD, Congenital heart disease. *For example, bronchopulmonary dysplasia. †Moderate to severe anemia, defined as hemoglobin < 90 g/L at hospital admission. ^a^*P* < 0.05, for comparison of median age in 2019–2020 with other groups; ^b^*P* < 0.05, for comparison of median age in 2020–2021 with other groups; ^c^*P* < 0.05, for comparison of median age in 2021–2022 with other groups (exclude 2020–2021)

The annual incidence of RSV from 2013 to 2014 to 2021–2022 was 15.8% (*n* = 839/5,304), 12.5% (*n* = 797/6,366), 11.3% (*n* = 656/5,786), 12.3% (*n* = 811/6,617), 14.9% (*n* = 831/5,595), 15.8% (*n* = 1,121/7,104), 13.1% (*n* = 596/4,561), 24.6% (*n* = 1,060/4,305), and 21.3% (*n* = 855/4,020), respectively. The highest rate occurred in 2020–2021, followed by 2021–2022 (Fig. 2).

**Fig. 4:**
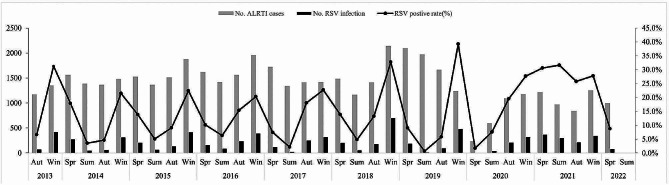
Seasonality distribution of RSV-positive specimens in children with ALRTI in Hunan from July 1,2013 to June 30, 2022. Abbreviation: RSV, respiratory syncytial virus

RSV can be detected throughout the year. During the study period, the overall detection rate was highest in winter (26.7%) and lowest in summer (6.5%). Seasonal variation was evident during different years. During 2016–2017, 2017–2018, and 2018–2019, RSV was detected most frequently in winter, followed by autumn, and the detection rate was lowest in summer. In February 2020, at the onset of the COVID-19 pandemic, the RSV detection rate suddenly dropped. Only four cases were detected in the spring of 2020, marking the lowest number in recent years (RSV-positive rate of 1.8%). From the summer of 2020 onward, the RSV detection rate remained consistently high, reaching an unprecedented peak in the summer of 2021 that persisted until the end of spring 2022 (Fig. 4).

The age of RSV-positive cases (*P* = 0.025), age distribution, average length of hospital stays, PICU admission rate, proportion of severe pneumonia cases, and hospitalization expenses exhibited significant differences among study years (Table 2). The percentage of children that need PCIU and severe pneumonia cases present a trend of reduction during the pandemic. Interestingly, the age distribution of RSV-positive children differed between 2020 and 2021 and 2021–2022, with a significantly greater number of positive cases in the 24–59 months age group compared to the < 6 months age group. The median age of infected children was 14 and 15 months for the periods of 2020–2021 and 2021–2022, respectively, with the median age significantly lower from 2013 to 2014 to 2019–2020.

**Fig. 5:**
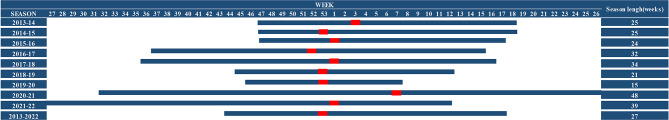
Timing of RSV epidemic seasons (deep blue) by calendar week from 2013 to 2022 in Hunan, China. The peak week is colored in red. The onset and offset weeks of RSV seasons according to different calculative methods. RSV epidemic season was defined as consecutive weeks during which the percentage of RSV-specific testing positive per week exceeded a 10% threshold. Abbreviation: RSV, respiratory syncytial virus

### RSV infection trends

**Fig. 6:**
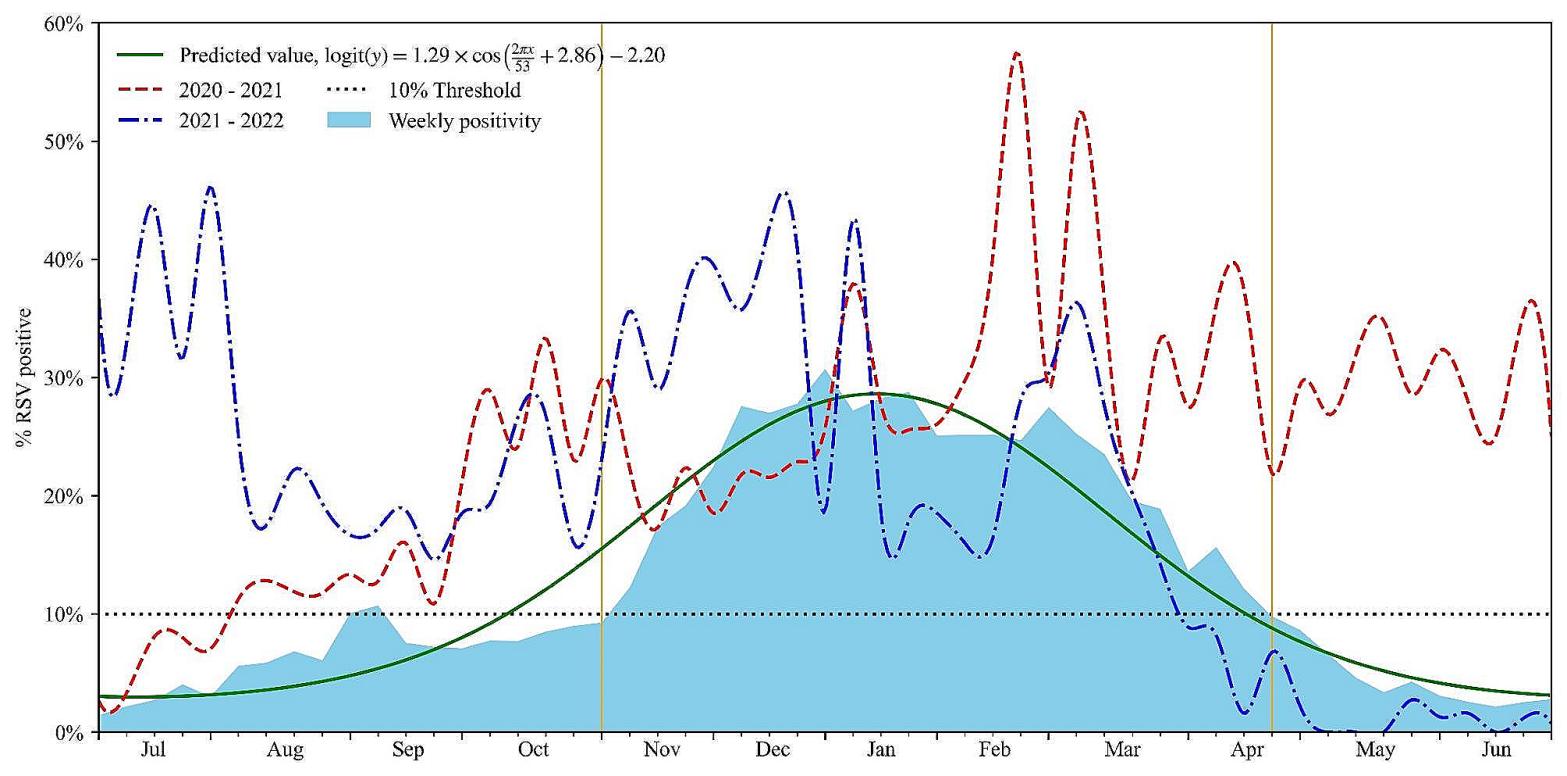
The predictions of year effect on the weeks of the average percentage of respiratory virus antigen tests positive for RSV per week in 2013–2022, as obtained from the non-linear ordinary least squares regression model. The graph begins at calendar week 27. Season onset and offset are indicated by the 2 orange vertical lines. The non-linear ordinary least squares regression model indicated that the year effect on the weeks of epidemic clusters was statistically significant (*P*-value < 0.001, Fig. 6). Thus, the epidemic season has changed. RSV activity in 2020–2021, resurgence during the summer and autumn, started earlier by 12 weeks when compared with the predictions of year, peaked later (the 7 week), and lasted longer. RSV activity in 2021–2022, started earlier by 18 weeks when compared with the predictions of year, peaked and offset within the predicted range

The monitoring year was determined based on the trough in the RSV cycle. Between 2013 and 2022, the surveillance year commenced on July 1 (27th week of epidemiology) and concluded on June 30 (26th week) of the subsequent year. Using our model, we identified nine distinct RSV seasons during the study period. The R^2^ value for our curve-fitting model, utilizing RSV data, exceeded 0.95. This indicated the effectiveness of the cosine model in predicting and evaluating the seasonal characteristics of RSV in Hunan. Based on a 10% cut-off point and the fitted seasonal curve, we analyzed various parameters for each of the 9 years: the start of the RSV season (the initial 2 consecutive weeks with an RSV-positive rate > 10%), the duration of the season, the peak week, the end of the season, and the proportion of RSV-positive cases (Figs. 5 and 6).

Figure 5 presents the weeks of the epidemic clusters of RSV infection detected per season. During 2013–2014, 2014–2015, and 2015–2016, the RSV season commenced in late autumn and lasted for 24–25 weeks, concluding in late spring of the subsequent year. By contrast, the onset of the RSV season in 2016–2017 and 2017–2018 occurred 11–15 weeks earlier, beginning between late summer and early autumn and lasting for 32–34 weeks, concluding at the end of the following spring. The epidemic clusters of the 2018–2019 seasons were observed between week 45 of 2018 and week 12 of 2019 (November 4–March 23). RSV epidemics during the 6 surveillance years preceding the COVID-19 pandemic (2013–2019) typically commenced in October (week 44), peaked in December (week 53), and lasted a median of 27 weeks before concluding in April (week 17). Around 81.7% of the RSV-positive cases occurred during the official RSV season. Figure 6 demonstrates that the fitting curve was generally consistent with the actual trend in RSV cases.

However, cluster weeks during the 2019–2020 seasons were of short duration. Based on the 10% epidemic threshold, no seasonal RSV epidemic was observed during the spring of 2020 due to the COVID-19 pandemic and associated NPIs. By contrast, the 2020–21 epidemic commenced 12 weeks earlier (August 4), peaked in February (2021) and January (2022), and lasted for 87 weeks before concluding in March 2022. The peak percentage of RSV-positive results was higher than that during pre-pandemic seasons (Figs. 3 and 5, and 6). The COVID-19 pandemic significantly influenced the RSV season in Hunan, with a brief decline in incidence during the spring of 2020, followed by a rapid recovery in the summer of the same year.

## Discussion

 The present study explored the surveillance data for RSV-related ALRTI in Hunan, China, and assessed the seasonality of RSV. On average, the RSV season initiated around the calendar week 44 (mid-October) and spanned 27 weeks, concluding by week 17 of the following year (late April). This seasonal pattern was consistent with that reported in Beijing, China [7]. A global overview of RSV seasonality indicated that, in the majority of countries, the commencement, conclusion, and peak of RSV activity typically varied by only 1–3 weeks from season to season [20]. The United States exhibited RSV season patterns, beginning in October, peaking in December or early February, and lasting a median of 27–31 weeks before concluding in March-April, a pattern consistent with our study [21]. Another study found that some states (e.g. Colorado, Iowa, California in the 1990s) exhibited biennial patterns of alternating ‘‘early-big’’ epidemics in/around January of even-numbered years and ‘‘late-small’’ epidemics in/around February of odd-numbered years [6]. The impact of RSV subgroup on seasonal severity remains a subject of controversy [22]. Monitoring data for Beijing, China, revealed that RSV infection prevalence was highest in winter and spring among children in northern China from 2007 to 2015. China reported that RSV seasons occurred 3–5 weeks earlier and lasted 6 weeks longer in RSV subgroup A-dominant years compared to subgroup B-dominant years [7].

China, a northern hemisphere country, exhibits a well-established RSV circulation pattern with peak incidences in winter months and declines in early spring [7]. However, the emergence and spread of SARS-CoV-2 and subsequent mitigation measures have led to widespread social disruption, influencing the seasonal circulation patterns of respiratory viruses [9, 23–25]. In our study, the total number of children hospitalized for ALRTIs decreased significantly starting in 2020. Despite similar overall detection rates of RSV compared to before, RSV decreased significantly after the outbreak, reaching historically low levels in the spring of 2020. A previous study reported that the monthly incidence rate of first-time medically attended RSV infection in children aged 0–5 years followed a consistent seasonal pattern from 2010 to 2019. The seasonal variation disappeared in 2020 and returned in 2021 but started earlier and reached a historically high rate of 2182 cases per 10,000 000 person-days in November 2022 [5]. A similar trend has been observed in several countries [9, 25, 26]. During the COVID-19 era, the interaction between RSV and SARS-CoV-2 has been widely discussed, with a viral interference effect and the implementation of NPIs proposed as explanations for these findings [25–28]. During the 2009 influenza pandemic, where large-scale NPIs were not implemented, there was an associated delay in the onset of the RSV season by an average of 0.58 months [29]. In Hongkong, with the occurrence of pandemic influenza A (H1N1) 2009 virus during the traditional summer peak for RSV in 2009, the RSV summer peak was not observed. In March 2010, the spring peak returned, the summer peak was again absent subsequently, and abnormal early rise of RSV activity was observed in the winter of 2010 that remained until the summer of 2011 [30]. A previous study [27] suggested that rhinovirus may have delayed the introduction of the pandemic virus into Europe, and conversely, the pandemic virus may have interfered with RSV epidemics. In March 2019, an adenovirus outbreak in Hunan Province concluded the RSV season nearly 1 month earlier than in previous years. These findings collectively suggest potential interference between viruses.

As public health and social measures were gradually lifted, the resurgence of RSV became a significant concern, drawing attention from experts globally. Modeling studies in the US have initiated exploration into the impact of increased population susceptibility due to minimal RSV and influenza virus infections in 2020–2021 on the magnitude of subsequent seasons [31]. Lina et al. in Tokyo, Japan, also expressed similar concerns using an epidemic model [32]. The abnormal reemergence of RSV during the COVID-19 era was also observed during the summer months in 2021 in northern hemisphere countries, including America [21], Israel [28], England [9], Japan [33], and China (Beijing, Shanghai) [34, 35]. Compared to these countries and cities, the summer peak of RSV in the post-COVID-19 phase occurred earlier and persisted longer in Hunan, China. Following the nationwide lockdown on January 23, 2020, there was a drop in the RSV detection rate. Even after the easing of the national lockdown on May 10, 2020, RSV detection remained low compared to the previous year. Approximately 2 weeks after the concurrent reopening of state primary and secondary schools in early September, there was a sharp increase in RSV detections, lasting for 87 weeks and concluding in the spring of 2022. Through continuous surveillance, it was observed that the RSV detection rates surpassed the historical levels of 2013–2019. In addition, the median age of RSV-infected children during the recovery phase was higher, with a greater number of children aged 24–59 months.

Several factors contribute to RSV seasonality and resurgence. First, the NPIs was lifted or relaxed. With the unblocking of Wuhan on April 8, 2020, the implementation of the national NPIs policy and the gradual migration of the population may have promoted the spread of the RSV virus. Li et al. [36] suggested that the full reopening of schools was the predominant risk factor for RSV rebound, increasing the risk by as much as 23-fold. Second, immunity debt may also be one of the important reasons. In the absence of an RSV vaccine, partial and transient immunity is naturally and spontaneously achieved each year by two mechanisms: infection or transplacental transfer of maternal RSV antibodies. Because of a widespread lack of exposure to RSV, particularly in younger cohorts, resulting in the build-up of an increased pool of susceptible. We also found that the median age of RSV-infected children during the recovery phase was elder, with the number of children aged 24 to 59 months, which may be the cumulative effect of susceptible groups. Waning maternal immunity due to low RSV exposure and the consequent decrease in transplacental RSV antibody transfer may have contributed to increased RSV infections also. Third, other factors may also be involved. The infection pattern of RSV resurgence remains different from that prior to 2019 and importantly significantly higher, suggesting the possibility that COVID-19 or COVID-related risk factors including lasting impact on immune systems that may partially account for high RSV incidence rates in young children. The observed resurgence reported by Foley et al. occurred following months of relaxed social distancing measures (not immediately following relaxation of measures) [10]. The studies showed that, in the context of reinforced public health measures in adults, maintaining children’s communities open (with reinforcement of social distancing and mandatory face masks since 6 years old) had low impact on RSV infections. Other factors which are believed to influence the epidemiology of respiratory viruses such as temperature, humidity, crowding in school classrooms and viral co-infections or superinfections may also influence the chances of RSV epidemics. High temperatures decrease the risk for RSV rebound, with every 5 °C increase reducing the risk by 37%. The full reopening of schools could override the counter-effect of high temperatures, explaining the out-of-season RSV epidemics during the COVID-19 pandemic seen in our study. The increase in numbers and the change in median age suggest that the expanded cohort of RSV-naïve patients, including an increased number of older children coupled with waning population immunity [37], may have contributed to the resurgence. Our data underline the greater fragility of RSV control in the population: a slight relaxation of public health measures was concomitant to the resurgence of RSV in Hunan.

The present study observed a significant increase in RSV-related hospital admissions during 2020–21 and 2021–22 compared to preceding years. Despite the substantial surge in case numbers, the outcomes for hospitalized RSV cases during the COVID-19 outbreak appeared to be less severe. Analysis of data on ICU admission, and the incidence of severe pneumonia revealed a descend in the severity of ALRTI caused by RSV. Studies in Italy have reported that the severity of RSV-associated disease during the delayed season was comparable to that during the previous season based on respiratory support and PICU admissions [38]. Similarly, studies from Shanghai [34], Beijing [35], and Western Australia [12] have reported a decrease in the severity of the delayed RSV seasons during COVID-19 compared to previous seasons. Conversely, a study from the USA reported more severe RSV-related disease in infants during the COVID-19 pandemic [39]. As for the decrease in the severity of the disease in this study, it may be related to the older median age of infected children, who have larger airways and better immune function. In addition, the descent of co-infection may also help to reduce the severity of the disease.

This study also had some limitations. First, it focused exclusively on ALRTI inpatients who had undergone a nasopharyngeal examination. Second, it is a single-center study, and it would be more convincing if the data were from different centers. Third, we did not analyze the link between other viral or bacterial co-infections and RSV resurgence. The circulation of multiple respiratory viruses may have resulted in a high level of interactions between viruses, including an increase in viral co-infections or superinfections. In addition, RSV typing was absent and we failed to explore the mechanism of changes in clinical characteristics and epidemic trend of RSV infection after the epidemic, which was limited by our laboratory testing methods and the fact that the study was retrospective.

## Conclusions

We enrolled 49,658 hospitalized children diagnosed with ALRTIs over a 9-year period, spanning both before and during the COVID-19 epidemic, to assess RSV prevalence. COVID-19 has influenced the transmission pattern of RSV since 2020, and the patients in our study exhibited distinct demographic and clinical changes in the context of COVID-19. RSV seasonality was disrupted during the COVID-19 pandemic, and the season exhibited an unusually prolonged duration. Despite these observations, RSV still warrants great attention to prevent unusual rebounds and unexpected impacts. In addition to future investigations and the development of passive and active immunization, thorough surveillance of RSV variation remains crucial as we transition into the post-COVID-19 era.

### Electronic supplementary material

Below is the link to the electronic supplementary material.

Supplementary Material 1

Supplementary Material 2

Supplementary Material 3

## Data Availability

The datasets of the current study are available from the corresponding author on reasonable request.

## Abbreviations

RSV: Respiratory syncytial virus
ALRTIs: Acute lower respiratory tract infections
COVID-19: The coronavirus disease 2019
NPIs: Non-pharmaceutical interventions
IQR: Interquartile ranges
SARS-CoV-2: Severe acute respiratory syndrome coronavirus 2
