# Correction: Seasonality of respiratory syncytial virus infection in children hospitalized with acute lower respiratory tract infections in Hunan, China, 2013–2022

**DOI:** 10.1186/s12985-024-02340-y

**Published:** 2024-03-20

**Authors:** Le-Yun Xie, Tao Wang, Tian Yu, Xian Hu, Le Yang, Li-Li Zhong, Bing Zhang, Sai-Zhen Zeng

**Affiliations:** Hunan Provincial People’s Hospital (The First Affiliated Hospital of Hunan Normal University),, Changsha, 410005 China

**Correction: Virology Journal (2024) 21:62** 10.1186/s12985-024-02336-8

Following publication of the original article [1], we have been notified that Fig. 5 was a duplicate of Fig. 6. Figure 5 has now been replaced with the correct version.

The original article has been corrected.
