# Supplementary material for: Seasonality of respiratory syncytial virus infection in children hospitalized with acute lower respiratory tract infections in Hunan, China, 2013–2022
Source: Virol J. 2024 Mar 7;21:62. doi: 10.1186/s12985-024-02336-8 (PMC10921640; doi:10.1186/s12985-024-02336-8)
Supplement: Supplementary file 1 — Supplementary Material 1 [file 12985_2024_2336_MOESM1_ESM.docx]

Supplementary table 1 Infection situation of 7 common respiratory viruses from July 1,2013 to June 30, 2022 in Hunan, China

|  |  | respiratory virus positive（n，%） | | | | | | | |
| --- | --- | --- | --- | --- | --- | --- | --- | --- | --- |
| Year | ALRTI tested for RSV(n) | RSV  No. (%) | HAdV  No. (%) | IFVA  No. (%) | IFVB  No. (%) | PIV-1  No. (%) | PIV-2  No. (%) | PIV-3  No. (%) | Total  No. (%) |
| 2013-2014 | 5304 | 839(15.8) | 88(1.7) | 126(2.4) | 22(0.4) | 104(2.0) | 31(0.6) | 289(5.4) | 1498(28.2) |
| 2014-2015 | 6366 | 797(12.5) | 47(0.7) | 94(1.5) | 55(0.9) | 13(0.2) | 4(0.1) | 240(3.8) | 1230(19.3) |
| 2015-2016 | 5786 | 656(11.3) | 43(0.7) | 323(5.6) | 203(3.5) | 39(0.7) | 20(0.3) | 388(6.7) | 1668(28.8) |
| 2016-2017 | 6617 | 811(12.3) | 182(2.8) | 223(3.4) | 91(1.4) | 55(0.8) | 10(0.2) | 317(4.8) | 1720(26.0) |
| 2017-2018 | 5595 | 831(14.9) | 126(2.3) | 221(3.9) | 128(2.3) | 31(0.6) | 36(0.6) | 314(5.6) | 1679(30.0) |
| 2018-2019 | 7104 | 1121(15.8) | 447(6.3) | 221(3.1) | 113(1.6) | 78(1.1) | 38(0.5) | 421(5.9) | 2441(34.4) |
| 2019-2020 | 4561 | 596(13.1) | 169(3.7) | 73(1.6) | 52(1.1) | 55(1.2) | 51(1.1) | 151(3.3) | 1147(25.1) |
| 2020-2021 | 4305 | 1060(24.6) | 19(0.4) | 45(1.0) | 40(0.9) | 322(7.5) | 4(0.1) | 118(2.7) | 1608(37.4) |
| 2021-2022 | 4020 | 855(21.3) | 27(0.7) | 70(1.7) | 93(2.3) | 13(0.3) | 11(0.3) | 225(5.6) | 1294(32.2) |
| Total | 49658 | 7566(15.2) | 1148(2.3) | 797(1.6) | 797(1.6) | 710(1.4) | 205(0.4) | 2463(5.0) | 14285(28.8) |

Notes: RSV indicate respiratory syncytial virus; Flu A indicate influenza virus A; Flu B indicate influenza virus B; ADV indicate adenovirus; PIV-1 indicate para-influenza virus 1; PIV-2 indicate para-influenza virus 2; and PIV-3 indicate para-influenza virus 3.
