# Supplementary material for: Seasonality of respiratory syncytial virus infection in children hospitalized with acute lower respiratory tract infections in Hunan, China, 2013–2022
Source: Virol J. 2024 Mar 7;21:62. doi: 10.1186/s12985-024-02336-8 (PMC10921640; doi:10.1186/s12985-024-02336-8)
Supplement: Supplementary file 2 — Supplementary Material 2 [file 12985_2024_2336_MOESM2_ESM.docx]

**Supplementary table 2**

**Distribution of different combinations of the 7566 RSV-ALRTI cases with pathogen co-infections**

| Co-infected viruses | No. | constituent ratio(%) |
| --- | --- | --- |
| RSV+ADV | 229 | 43.6 |
| RSV+PIV3 | 64 | 12.2 |
| RSV+IFVA | 61 | 11.6 |
| RSV+IFVB | 50 | 9.5 |
| RSV+PIV1 | 46 | 8.8 |
| RSV+PIV2 | 6 | 1.1 |
| RSV+ADV+IFVA | 35 | 6.7 |
| RSV+ADV+IFVB | 6 | 1.1 |
| RSV+ADV+PIV1 | 3 | 0.6 |
| RSV+ADV+PIV2 | 2 | 0.4 |
| RSV+ADV+PIV3 | 2 | 0.4 |
| RSV+IFVA+IFVB | 6 | 1.1 |
| RSV+IFVA+PIV1 | 2 | 0.4 |
| RSV+IFVA+PIV2 | 2 | 0.4 |
| RSV+IFVA+PIV3 | 2 | 0.4 |
| RSV+IFVB+PIV3 | 1 | 0.2 |
| RSV+PIV1+PIV3 | 1 | 0.2 |
| RSV+PIV2+PIV3 | 1 | 0.2 |
| RSV+ADV+IFVA+IFVB | 4 | 0.8 |
| RSV+ADV+PIV1+PIV2 | 1 | 0.2 |
| RSV+ADV+IFVA+PIV1+PIV2 | 1 | 0.2 |
| Total | 525 | 1 |

Notes: RSV indicate respiratory syncytial virus; Flu A indicate influenza virus A; Flu B indicate influenza virus B; ADV indicate adenovirus; PIV-1 indicate para-influenza virus 1; PIV-2 indicate para-influenza virus 2; and PIV-3 indicate para-influenza virus 3.
