# Supplementary material for: Seasonality of respiratory syncytial virus infection in children hospitalized with acute lower respiratory tract infections in Hunan, China, 2013–2022
Source: Virol J. 2024 Mar 7;21:62. doi: 10.1186/s12985-024-02336-8 (PMC10921640; doi:10.1186/s12985-024-02336-8)
Supplement: Supplementary file 3 — Supplementary Material 3 [file 12985_2024_2336_MOESM3_ESM.docx]

**Title: Seasonality of respiratory syncytial virus infection in children hospitalized with acute lower respiratory tract infections in Hunan, China, 2013–2022**

**Running title: RSV seasonality changed**

**Author：**

Le-Yun Xie^1#^, Tao Wang^1#^, Tian Yu^1 *^, Xian Hu^1^, , Le Yang^1^, Li-Li Zhong^1^，Bing Zhang^1^ , Sai-Zhen Zeng^1 *^

M.D. Le-Yun Xie 258137134@qq.com

M.D. Tao Wang 1090432322@qq.com

M.D. Tian Yu csyt77@126.com

M.D. Xian Hu 542613744@qq.com

M.D. Le Yang 10013481@qq.com

M.D. Li-Li Zhong 570047414@qq.com

M.D. Bing Zhang zhangbing1959@aliyun.com

M.D. Sai-Zhen Zeng 55445321@qq.com

**Address**

^1^ Hunan Provincial People’s Hospital (The First Affiliated Hospital of Hunan Normal University), Changsha 410005, China

**# These authors contributed equally to this work and should be considered co-first authors.**

***Co-corresponding author**

Sai-Zhen Zeng, M.D.

Hunan provincial People’s Hospital (The First Affiliated Hospital of Hunan Normal University).

61 Jie-Fang west road, Fu-Rong District, Changsha 410005, China.

Fax: 86-0731-8227, 8012

E-mail: [55445321@qq.com](mailto:55445321@qq.com)

Tian Yu, M.D

Hunan Provincial People’s Hospital (The First Affiliated Hospital of Hunan Normal University).

61 Jie-Fang west road, Fu-Rong District, Changsha 410005, China.

Fax: 86-0731-8227, 8012

1. mail: [csyt77@126.com](mailto:csyt77@126.com)
